# Does cystic fibrosis constitute an advantage in COVID-19 infection?

**DOI:** 10.1186/s13052-020-00909-1

**Published:** 2020-10-06

**Authors:** Valentino Bezzerri, Francesca Lucca, Sonia Volpi, Marco Cipolli

**Affiliations:** Cystic Fibrosis Center of Verona, Azienda Ospedaliera Universitaria Integrata, 37126 Verona, Italy

**Keywords:** Cystic fibrosis, SARS-CoV-2, Covid-19, Azythromycin, DNase

## Abstract

The Veneto region is one of the most affected Italian regions by COVID-19. Chronic lung diseases, such as chronic obstructive pulmonary disease (COPD), may constitute a risk factor in COVID-19. Moreover, respiratory viruses were generally associated with severe pulmonary impairment in cystic fibrosis (CF). We would have therefore expected numerous cases of severe COVID-19 among the CF population. Surprisingly, we found that CF patients were significantly protected against infection by SARS-CoV-2. We discussed this aspect formulating some reasonable theories.

## Introduction

The comorbidities of obesity, hypertension, diabetes, heart failure, and chronic lung disease have been associated with poor outcome in coronavirus disease 2019 (COVID-19) [1]. Once Severe Acute Respiratory Syndrome (SARS)– Coronavirus (CoV)-2 has infected host cells, excessive inflammatory and thrombotic processes take place. A cytokine storm release with markedly elevated IL-6 levels are associated with increased lethality [2]. Increased neutrophilia is part of that inflammatory response and has been associated with a poorer outcome. Increased neutrophil extracellular traps (NET) have been hypothesized to be predictive of poor outcome in COVID-19 patients [3].

Cystic fibrosis (CF) lung disease, characterized by the release of a many proinflammatory cytokines and chemokines, is the major cause of morbidity and mortality in CF. Excessive NET accumulation has been observed in the CF airway [4]. Moreover, influenza and other respiratory viral infections are known to cause severe pulmonary impairment in CF. [5] Because of the risk factors of chronic lung disease and host hyper-immune status, one would surmise that CF patients would be at an increased risk of developing severe COVID-19 illness.

## Methods

We conducted a retrospective study of 532 CF patients followed at the Cystic Fibrosis Center of Verona, Italy. SARS-CoV-2 positivity was tested by collecting combined nose-throat swabs and subsequent Real-Time PCR using the Nimbus MuDT tm (Seegene, Seoul, South Korea), and Simplexa COVID-19 Direct kits (DiaSorin, Stillwater, MN, USA), following the manufacturer guidelines. All patients tested in this study reported symptoms consistent with respiratory infection and were hospitalized at Cystic Fibrosis Center of Verona.

## Results

The Veneto region is one of the Italian regions most strongly affected by COVID-19, with 19,729 cases and 2062 related fatalities (as of 23 July 2020). The Veneto region health system has carried out 1,149,084 multiple COVID-19 tests on 465,433 subjects so far (data from the Italian Ministry of Health).

From 1 April to 23 July 2020, we contacted all the 532 patients from Veneto, of whom 118 subjects (22.2%), with a median age of 32.4 years (range 0.12–65.3 years), undergone further combined nose and throat swab test for SARS-CoV-2. Surprisingly, we found that CF patients were significantly protected against infection by SARS-CoV-2. We identified only one CF patient, a 46-year-old man, who underwent lung transplantation in 2011, with COVID-19 infection. Prior to COVID-19 positivity, this patient exhibited a preserved lung function (mean ppFEV1 in the last 12 months 90.4%), MS-*Staphylococcus aureus* and *Pseudomonas aeruginosa* colonization of the airways, chronic kidney disease, impaired nutritional status (BMI 17.7 kg/m^2^). Upon SARS-CoV-2 infection, the patient reported only mild symptoms including dry cough and body temperature, and he did not require intensive care unit (ICU) admission. These data indicate that the COVID-19 infection rate in the Veneto regional CF population is 0.19%, in contrast to 0.40% in the general population (Table 1).

**Table 1:** Demographic and Epidemiologic Data from the Veneto Region

Condition	Population	Mean (years)	Tested subjects (% of regional population)	COVID-19 cases	Rate of infection (% of regional population)	COVID-19 fatalities	CFR
General population	4,907,704^a^	45,4	465,433 (9.5%)	19,729	0.40%	2062	10.4%
CF population	532	27,5	118 (22.2%)	1	0.19%	0	–

*CFR* case fatality rate

^a^Data from the Istituto Nazionale di Statistica (ISTAT) of Italy, updated to 1 January 2020

## Discussion

CF condition is supposed to represent a comorbidity for COVID-19. Our data show instead that the prevalence of COVID-19 in CF population of one of the Italian region most affected by the SARS-CoV-2 outbreak, namely Veneto region, is reduced compared with general population. Interestingly, our data are in line with emerging evidence observed by the “COVID-CF project in Europe” of the European Cystic Fibrosis Society (https://www.ecfs.eu/covid-cf-project-europe). According to preliminary data from this project, among approximately 48,000 European CF patients, eight patients needed ICU admission and only three died for COVID-19 (CFR 3.2%) (as of 15 July 2020).

Several factors might play a role in reducing the prevalence and, perhaps, the lethality of COVID-19: typical behavior in patients with CF, who might wear protective masks and avoid contact with sick people, use of antibiotics, or host factors. A more mild form of COVID-19 might be expected because disruption of IL-6 signaling in CF lungs occurs through elevated serine protease release and subsequent cleavage of both membrane-bound and soluble IL-6 receptors [6]. Chronic pharmacological therapies, including azithromycin (immunomodulator) and DNase (mucolytic), administered to 56 and 51% of our patients, respectively, might also contribute to the protection. Vitamin D deficiency is a common feature of CF condition. The result is that CF patients generally undergo vitamin D supplementation, which may have a protective effect against COVID-19. However, it has been reported that the levels of vitamin D in CF patients remain lower than normal even after supplementation [7].

All together, these data strengthen the hypothesis that CF may constitute an advantage in COVID-19, encouraging the scientific community to look for the possible reasons behind this unexpected scenario.

## Data Availability

Data were obtained from the Institutional Database at Azienda Ospedaliera Universitaria Integrata, Verona.

## Abbreviations

CF: Cystic Fibrosis
ICU: intensive care unit
SARS-CoV-2: Severe Acute Respiratory Syndrome-Coronavirus-2
COVID-19: Coronavirus disease 2019
NET: Neutrophil extracellular trap
